# Optimal COVID-19 therapeutic candidate discovery using the CANDO platform

**DOI:** 10.3389/fphar.2022.970494

**Published:** 2022-08-25

**Authors:** William Mangione, Zackary Falls, Ram Samudrala

**Affiliations:** Department of Biomedical Informatics, Jacobs School of Medicine and Biomedical Sciences, University at Buffalo, Buffalo, NY, United States

**Keywords:** COVID-19, SARS-CoV-2, drug discovery, multitargeting, computational drug repurposing, computational biology

## Abstract

The worldwide outbreak of SARS-CoV-2 in early 2020 caused numerous deaths and unprecedented measures to control its spread. We employed our Computational Analysis of Novel Drug Opportunities (CANDO) multiscale therapeutic discovery, repurposing, and design platform to identify small molecule inhibitors of the virus to treat its resulting indication, COVID-19. Initially, few experimental studies existed on SARS-CoV-2, so we optimized our drug candidate prediction pipelines using results from two independent high-throughput screens against prevalent human coronaviruses. Ranked lists of candidate drugs were generated using our open source cando.py software based on viral protein inhibition and proteomic interaction similarity. For the former viral protein inhibition pipeline, we computed interaction scores between all compounds in the corresponding candidate library and eighteen SARS-CoV proteins using an interaction scoring protocol with extensive parameter optimization which was then applied to the SARS-CoV-2 proteome for prediction. For the latter similarity based pipeline, we computed interaction scores between all compounds and human protein structures in our libraries then used a consensus scoring approach to identify candidates with highly similar proteomic interaction signatures to multiple known anti-coronavirus actives. We published our ranked candidate lists at the very beginning of the COVID-19 pandemic. Since then, 51 of our 276 predictions have demonstrated anti-SARS-CoV-2 activity in published clinical and experimental studies. These results illustrate the ability of our platform to rapidly respond to emergent pathogens and provide greater evidence that treating compounds in a multitarget context more accurately describes their behavior in biological systems.

## 1 Introduction

The severe acute respiratory syndrome coronavirus 2 (SARS-CoV-2) and the disease caused by its infection, COVID-19, was first documented in Wuhan, China in December 2019. It spread rapidly and was declared a pandemic by the World Health Organization in March 2020, causing over 5.9 million deaths across the world as of February 2022 (Organization, 2022). The scientific community immediately began employing various tools and methods to identify medical interventions that would reduce the threat posed by this novel coronavirus. Numerous institutions conducted clinical trials evaluating the ability of therapeutics to decrease COVID-19 lethality, often reporting conflicting results for the same drug (e.g. chloroquine and remdesivir) (Wang Y. et al., 2020; Chowdhury et al., 2020; Spinner et al., 2020). Few clearly conclusive success stories were reported in the months immediately following the outbreak with the most notable being dexamethasone, an anti-inflammatory corticosteroid that reduced death rates in patients suffering from a hyperactive immune system response known as a cytokine storm (Group, 2021). Further, it took nearly two years for a direct antiviral therapeutic indisputably capable of significantly preventing death from COVID-19 to be approved by the FDA, specifically both molnupiravir and the nirmatrelvir/ritonavir combination drugs in December of 2021 (Mahase, 2021; Hammond et al., 2022), which speaks to the complexity of this disease and the urgent need for innovative technologies that rapidly and effectively identify promising therapies. Such technologies will not only be useful in the present but also to combat any new emerging pathogens.

Significant advances made in the field of computational drug discovery were deployed in the context of COVID-19 with the goal of uncovering viable solutions (Mohamed et al., 2021). For example, multiple studies utilized virtual docking methods to identify compounds with strong affinity to SARS-CoV-2 proteins (Vijayan et al., 2020; Wang, 2020; Baby et al., 2021). Others used network-based bioinformatics methods to suggest drug repurposing candidates or better understand SARS-CoV-2 pathology, taking advantage of large scale human and virus protein-protein interaction knowledge (Zhou et al., 2020; Ghandikota et al., 2021; Gysi et al., 2021). On the clinical side, applications of traditional and deep machine learning methods have been utilized to identify high-risk patients, such as convolutional neural networks that analyze CT and X-ray images (Ardakani et al., 2020; Ozturk et al., 2020). Deep learning approaches have also been directly applied to identify drug candidates for treating COVID-19 (Liu et al., 2021; Pham et al., 2021).

In this study we describe and evaluate the performance of our Computational Analysis of Novel Drug Opportunities (CANDO) multiscale therapeutic drug discovery, repurposing, and design platform for identifying small molecules that show potential in inhibiting the SARS-CoV-2 virus and treating COVID-19. CANDO was originally designed as a shotgun repurposing platform for exactly this type of epidemic/pandemic scenario utilizing multiscale modeling techniques and adhering to multitarget drug theory, but has since been enhanced to carry out novel drug discovery against all indications (Jenwitheesuk and Samudrala, 2003b, 2005; Jenwitheesuk et al., 2008; Horst et al., 2012; Minie et al., 2014; Sethi et al., 2015; Chopra et al., 2016; Chopra and Samudrala, 2016; Falls et al., 2019; Fine et al., 2019; Mangione and Samudrala, 2019; Schuler et al., 2019; Schuler and Samudrala, 2019; Mangione et al., 2020b; Hudson and Samudrala, 2021; Schuler et al., 2021) as well as novel drug design (Overhoff et al., 2021). The relatively recent introduction of higher order biological data such as protein pathways, protein-protein interactions, drug side effects, and protein-disease associations has further augmented our ability to describe compound behavior holistically, with subsequent improved performance (Moukheiber et al., 2021; Schuler et al., 2021; Mangione, 2022; Mangione et al., 2022). Our platform is freely available to the scientific community and a detailed description of the software implementation has been published (Mangione et al., 2020a).

We employed two separate predictive pipelines within CANDO to suggest putative drug candidates for COVID-19: one first optimized our compound-protein interaction protocol against SARS-CoV and then applied it to SARS-CoV-2, and the other searched for compounds that were similar to those known to possess anti-coronavirus activity based on interactions computed with all human proteins. We originally published three different ranked lists of putative drug candidates in March and May of 2020 using the CANDO platform (Mangione et al., 2020b; Group, 2020). In May 2020, we published an assortment of drug candidates that were highly ranked by CANDO and were at the time being investigated in clinical trials to treat COVID-19. Since then several of our top scoring compounds have been validated by us and by others which we analyze in detail here. The significant number of top-ranked therapeutics successfully validated in this study, our previous work with the Ebola Virus Disease outbreak in West Africa in 2014 (Chopra et al., 2016), as well as our earlier validation studies and analyses (Jenwitheesuk and Samudrala, 2003b,a, 2005; Jenwitheesuk et al., 2008; Costin et al., 2010; Nicholson et al., 2011; Michael et al., 2011a,b), all suggest that CANDO is an effective tool to combat newly emerging epidemics and pandemics.

## 2 Results and discussion

**FIGURE 1:**
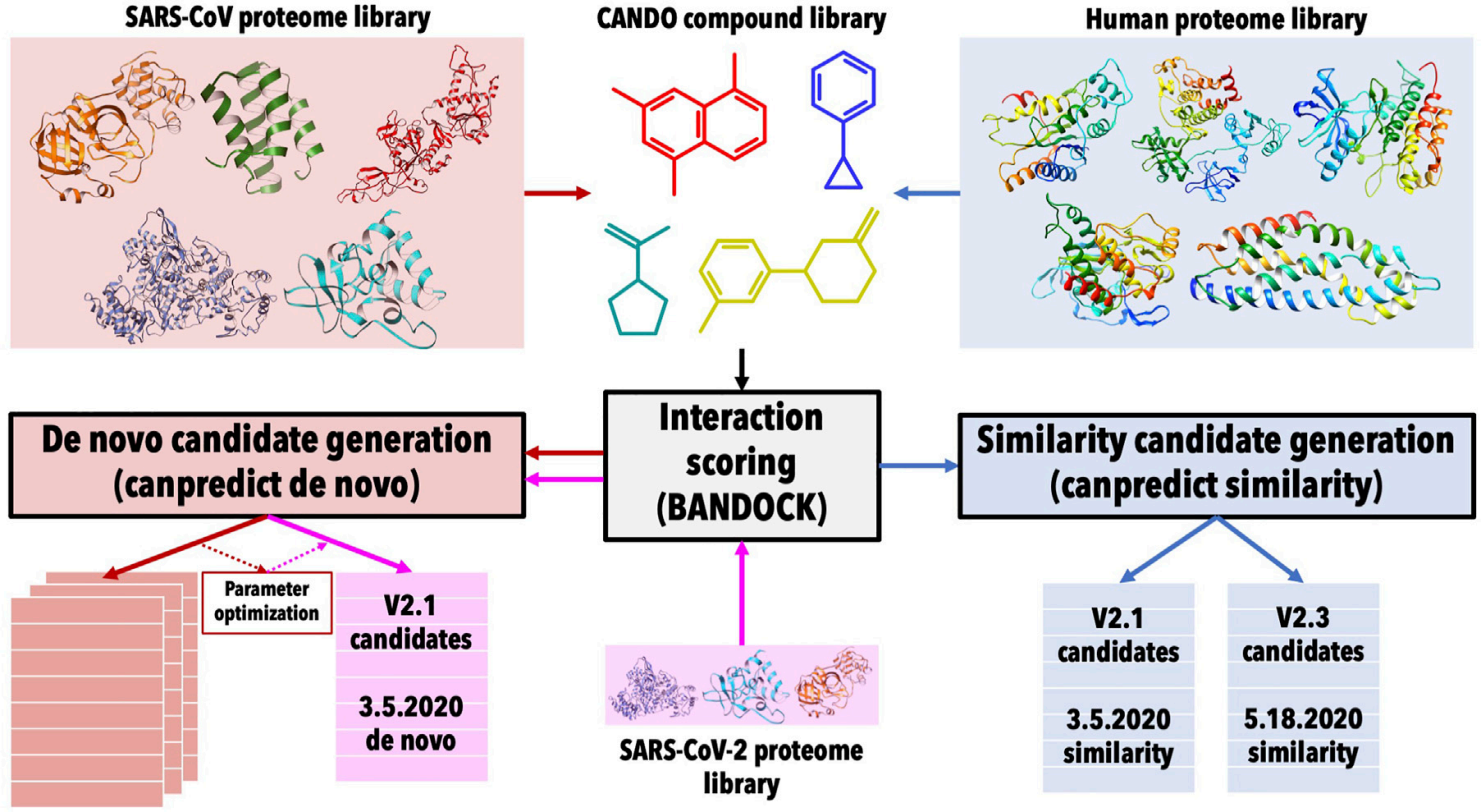
Overview of COVID-19 drug candidate prediction pipelines within the CANDO platform. Drug/compound structure libraries were curated from DrugBank (Wishart et al., 2018) and protein structure libraries comprising both the human and SARS-CoV proteomes were extracted from the Protein Data Bank (Burley et al., 2019). Interaction scores between every protein and compound in the corresponding libraries were calculated using our bioanalytic docking (BANDOCK) protocol (Mangione et al., 2020a; Schuler et al., 2021). The interaction scores with the SARS-CoV proteins were used for the *de novo* candidate generation pipeline (red) that identified compounds with the highest binding scores to multiple viral proteins, while the interaction scores with the human proteins were used for a similarity based candidate generation pipeline (blue) that identified candidates based on the similarity of their proteomic interaction signatures to drugs/compounds known to be effective against SARS-CoV *in vitro*. The interaction scoring protocol parameters were optimized against SARS-CoV and then applied to modeled protein structures from the SARS-CoV-2 proteome in the *de novo* candidate generation pipeline to produce the 3.5.20 *de novo* candidate list. Two distinct signature similarity drug candidate lists were generated using the version 2.1 CANDO compound library initially followed by an enhanced v2.3 compound library denoted as 3.5.20 similarity and 5.18.20 similarity, respectively. The predictions in these three lists were validated using evidence from published clinical and experimental studies to not only verify our platform but to determine optimal candidates that are safe and effective at treating COVID-19 downstream.

Figure 1 illustrates the pipelines and protocols used within the CANDO platform to produce the three lists of drug candidates; a detailed description follows below.

### 2.1 Compound-protein interaction protocol parameter optimization

We initially assessed the robustness of predictions made by the CANDO platform by inspecting the recapture rate of small molecules identified to be active against SARS-CoV, MERS-CoV, and other coronavirus species from two high-throughput screens by Shen et al. and Dyall et al. (Dyall et al., 2014; Shen et al., 2019).

**FIGURE 2:**
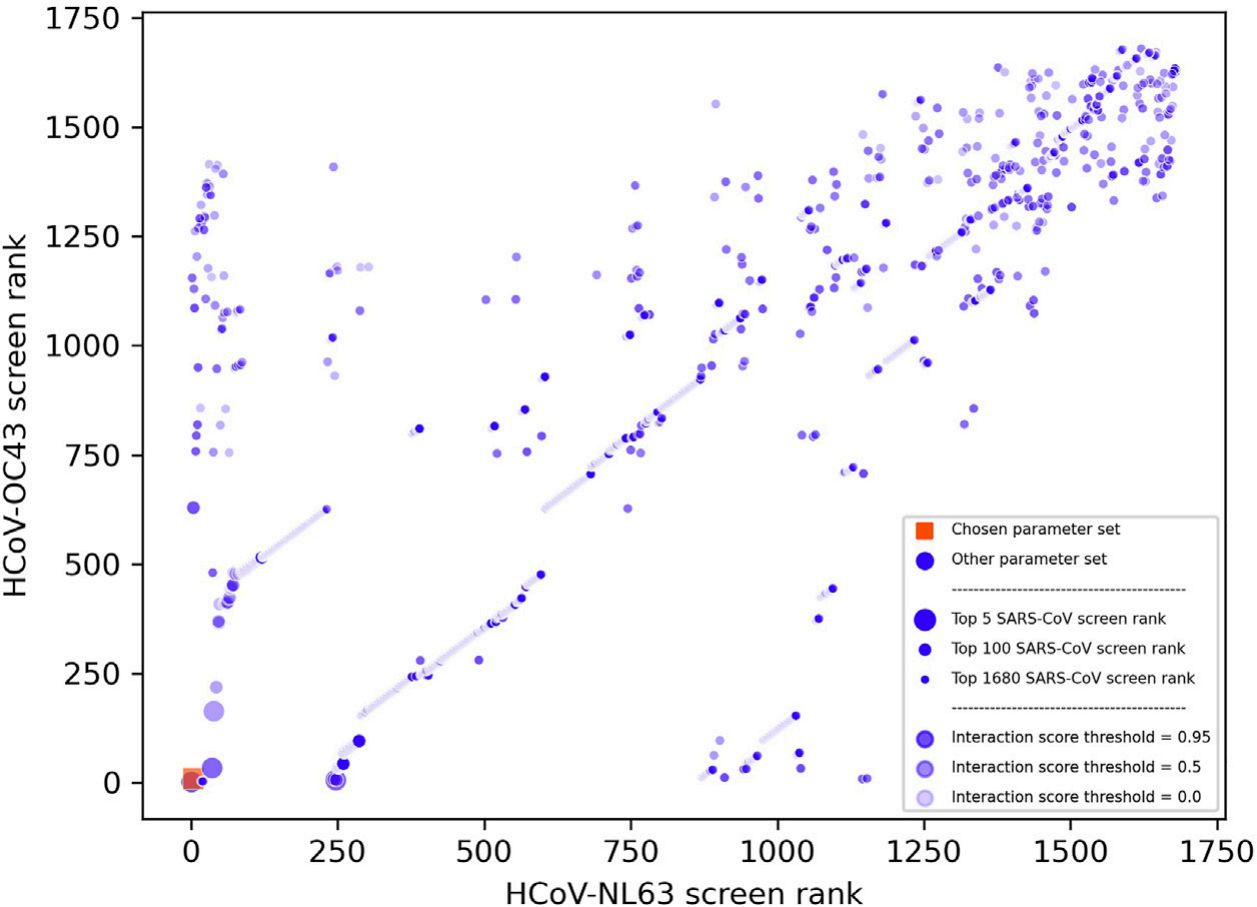
Visualization of parameter optimization set ranks across three coronavirus screens. This scatter plot depicts the ranks of each set of parameters for the interaction scoring protocol and *de novo* candidate generation pipeline within CANDO after using the discounted cumulative gain metric to score how well each corresponding pipeline ranked sets of active compounds against three separate coronavirus species: HCoV-NL63, HCoV-OC43, and SARS-CoV. The ranks for the HCoV-NL63 and HCoV-OC43 screens are depicted along the horizontal and vertical axes, respectively, while the size of the points depicts if the screen against SARS-CoV ranked within the top 5, 100, or 1,680 for each parameter set. The shade reflects the interaction score threshold that was used by the *de novo* pipeline to filter the candidates, scaled continuously from 0.0 (lightest) to 0.95 (darkest). The chosen parameter set (orange box) was the second ranked among all three screens with ranks of 3, 10, and 25 for HCoV-NL63, HCoV-OC43, and SARS-CoV, respectively, and used an ECFP10 integer based fingerprint, dCxP scoring protocol, and 0.9 compound-protein interaction score cutoff. The strong and consistent performance of this parameter set across three different coronavirus species justified our selection and warranted its use in generating drug candidates to inhibit SARS-CoV-2.

We parameterized our compound-protein interaction scoring protocol *via* the discounted cumulative gain metric after generating many matrices using various criteria (see Section 3.4). Figure 2 depicts how well each parameter set ranked the actives present in the three separate screens. Among the top four competitive parameter sets, two did not have any screens ranked within the top 10 and were discarded. The parameter set we chose to apply to SARS-CoV-2 ranked 25th for SARS-CoV, 3rd for HCoV-NL63, and 10th for HCoV-OC43. We selected this over the other competitive parameter set because omacetaxine mepesuccinate, one of the strongest actives identified in the Dyall screen, was ranked 2nd versus being ranked 14th in the discarded set. The final interaction scoring protocol and corresponding de novo candidate generation pipeline parameters included the integer based Extended-connectivity fingerprint (ECFP) with a diameter of 10, dCxP scoring protocol, and a compound-protein interaction score cutoff of 0.9 (see Section 3.3 and Section 3.4).

### 2.2 Generation and validation of drug candidates

We generated three lists of drug candidates from corresponding pipelines that mixed and matched the protocols and data sources as described in the methods: 1) Using the parameters identified in the previous step, we generated a list of 155 approved drug candidates with strong interaction scores to SARS-CoV-2 proteins where the top scoring compounds all had interaction scores greater than or equal to 0.9 to one or both of the main (Mpro) or papain-like (PLpro) proteases (identified as 3.5.20 *de novo*). 2) The nonredundant synthesis of the 18 actives from the Shen study and 21 actives from the Dyall study as well as 2 promising manually added candidates oseltamivir and remdesivir served as input to the interaction signature similarity pipeline since it does not require EC50 values. These 38 compounds were then used to generate 45 approved drug candidates using the signature similarity pipeline (3.5.20 similarity). 3) We later repeated the similarity pipeline with a sublibrary of 85 anti-SARS-CoV-2 actives and an enhanced CANDO compound library (v2.3) to generate a list of 97 approved drug candidates (5.18.20 similarity).

**FIGURE 3:**
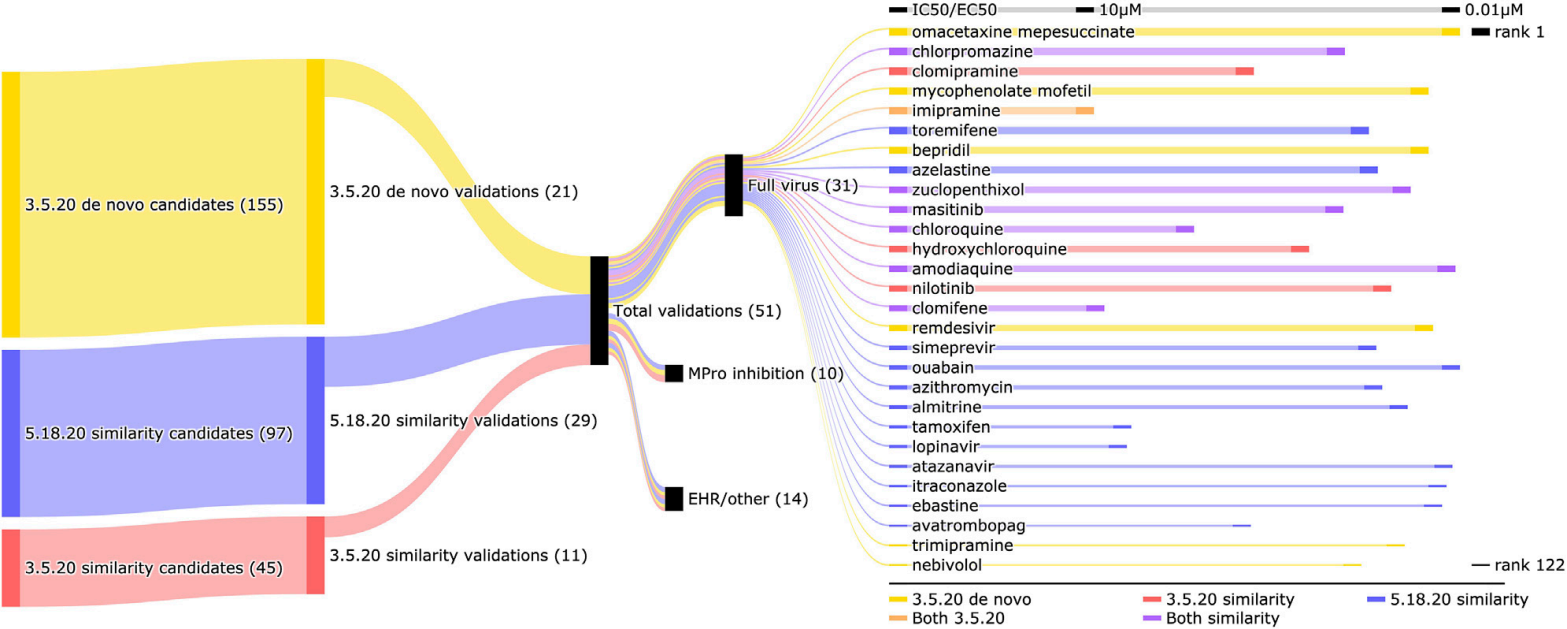
Validation of SARS-CoV-2 full virus inhibition candidates generated by the CANDO platform. The flow of validations among the three candidate lists generated using the CANDO platform are depicted from left to right using a Sankey diagram. The 155 candidates from 3.5.20 *de novo* (yellow) yielded 21 validations, while the similarity counterpart from the same date (red) produced 11 validations from 45 candidates. The 5.18.20 similarity list (blue) of 97 approved drugs resulted in 29 validations, resembling the hit rate of 3.5.20 similarity list and more than twice that of the lone *de novo* list. The 51 total validations were comprised of 31 full virus studies, 10 main protease (Mpro) inhibition studies, and 14 EHR or other inhibition based studies. The compounds that were validated *via* a full virus inhibition of less than 10 *μ*M are shown prioritized by their rank in the list (or best rank if in multiple lists) corresponding to the thickness of their bars (ranging from rank 1–122). All but six drugs were in a single list, five drugs were in both similarity based lists (purple) and one was in both the 3.5.20 similarity and *de novo* lists (orange). The length of the horizontal bar next to the names of the compounds indicates the lowest reported EC50 or IC50 from published experimental studies progressing on a linear scale. The second strongest reported EC50 (0.03 *μ*M) belongs to omacetaxine mepesuccinate, which is the top candidate from 3.5.20 *de novo*. The correlation between rank and strength of inhibition is sub-moderate (0.3718), and this is possibly due to the variation in assay design among different studies (viral replication reduction, viral entry inhibition, viral induced cytopathic effect reduction, etc). Overall, the CANDO platform was able to identify several candidates with potent anti-SARS-CoV-2 activity using two different predictive pipelines, verifying its potential to rapidly and efficiently respond to emerging threats to global health.

We scoured the literature to see if other studies validated our candidates from our three lists against SARS-CoV-2, primarily utilizing two different resources that collate detailed information on therapeutic interventions against COVID-19: CoronaCentral and the Targeting COVID-19 Portal from the Global Health Drug Discovery Institute (GHDDI) (see Section 3.6). Table 1 gives a summary of the number of predicted candidates and validations, along with correlation coefficients and discounted cumulative gain scores. Table 2 gives a full breakdown of the validations from each list as well as two drugs with weak EC50s not counted as validated: moxifloxacin and levofloxacin. This includes full virus, main protease, other miscellaneous *in vitro* (for example, inhibition of SARS-CoV-2 spike protein binding to the human ACE2 receptor), and electronic health record (EHR) studies. The studies demonstrating the activities are provided in Supplementary Table S1 while the energetic stability of the designated hits are provided in Supplementary Table S2. Figure 3 uses a Sankey diagram to illustrate the validation of all candidates with EC50s less than 10*μ*M, which includes 31 drugs that were found to be effective against SARS-CoV-2 in full virus inhibition studies. Overall, a total of 51 drugs showed efficacy against SARS-CoV-2 out of 275 nonredundant candidates for a hit rate of 18.5%.

**TABLE 1:** Summary details of drug candidates generated by the CANDO platform. For each candidate list, the total number of candidates that were initially generated by our prediction modules, the number of viable candidates after manual filtering (removing ions and dyes) prior to validation, the number of approved compounds, the number of candidates that were matched *via* literature search using the CoronaCentral and GHDDI resources (“Checked”), the number of candidates with EHR evidence or *in vitro* activity less than 100 *μ*M (“Validated”), the hit rate percentage, the Pearson correlation coefficient (“CC”) between the full virus validation ranks and their EC50 scores (including the combined and nonredundant lists), and the discounted cumulative gain (“DCG”) score are given. Overall, we obtained hit rates ranging from 13.5 to 29.9% using the CANDO platform, with the signature similarity pipelines yielding the highest success rates and the direct viral inhibition *de novo* pipeline accurately ranking the best, most potent, candidates.

	Total	Viable	Approved	Checked	Validated	Hit rate	CC	DCG
3.5.20 *de novo*	225	224	155	48	21	13.5%	0.41	0.96
3.5.20 similarity	115	114	45	17	11	24.4%	0.63	0.24
5.18.20 similarity	100	97	97	48	29	29.9%	0.35	0.22
Combined	440	435	297	113	61	20.5%	0.30	—
Nonredundant	419	414	275	102	51	18.5%	0.37	—

**TABLE 2:** Complete list of validated candidates generated by the CANDO platform. The names of the 51 compounds, their ranks in the 3.5.20 *de novo*, 3.5.20 similarity, and 5.18.20 similarity lists, the full virus EC50s, main protease IC50s, and EHR-based evidence are given. Only the lowest full virus EC50 for each candidate is shown. The *de novo* pipeline identified better, more potent, full virus inhibition candidates, while the signature similarity pipeline identified a greater fraction of validated candidates accurately.

Compound	3.5.20	3.5.20	5.18.20	SARS-CoV-2	Mpro IC50	Other
	*de novo*	similarity	similarity	EC50 (*μ*M)	(*μ*M)	
Omacetaxine mepesuccinate	1	—	—	0.03	—	—
Chlorpromazine		3	11	3.14	—	—
Clomipramine		4	—	5.63	—	—
Entrectinib	—	—	4		—	58.4 *μ*M IC50 Spike protein binding ACE2
Mycophenolate mofetil	7	—	—	0.87	—	—
Imipramine	127	8	—	10.0	—	—
Toremifene	—	—	8	2.5	—	—
Tamsulosin	100	14	38		—	18% relative risk reduction (death)
Bepridil	15	—	—	0.86	72	—
Azelastine	—	—	15	2.24	—	—
Zuclopenthixol	—	28	18	1.35	—	—
Masitinib	—	20	50	3.2	—	—
Erythromycin	—	—	20	—	—	70% reduction SARS-2 infection at 100ug/ml
Chloroquine	—	21	96	7.28	—	—
Ritonavir	—	—	21		13.7	—
Hydroxychloroquine	—	22		4.14	—	—
Cobicistat	—	—	22		6.7	—
Amodiaquine	—	23	40	0.13	—	—
Nilotinib	—	26	—	1.88	—	4.21 *μ*M IC50 Spike protein binding ACE2
Pimozide	—	—	26		42	—
Diphenhydramine	28	—	—	17.4	—	—
Clomifene	—	29	84	9.73	—	—
Remdesivir	30	—	—	0.76	—	—
Butenafine	—	—	35	—	5.4	—
Moxifloxacin	—	44	—	239.7	—	—
Clarithromycin	—	—	47	—	—	78% reduction in severe respiratory failure versus chloroquine
Saquinavir	—	—	54	—	9.92	—
Simeprevir	—	—	55	2.3	48.2	—
Ouabain	—	—	56	0.024	—	—
Azithromycin	—	—	57	2.12	—	—
Tranylcypromine	57	—	—	—	8.64	—
Almitrine	—	—	68	1.42	—	—
Tamoxifen	—	—	74	8.98	—	—
Colistimethate	—	—	75	—	—	Mpro 17% bound (50 *μ*M)
Lopinavir	—	—	76	9.12	—	—
Terconazole	144	—	78	11.92	—	—
Silodosin	81	—	—	—	—	18% relative risk reduction (death)
Atazanavir	—	—	82	0.22	60.7	—
Triamterene	86	—	—	—	—	23.5 *μ*M IC50 Spike protein binding ACE2
Hydroxyzine	90	—	—	15.3	—	0.42 hazard ratio (death)
Itraconazole	—	—	90	0.39	—	—
Ebastine	—	—	92	0.5	57	—
Avatrombopag	—	—	95	5.71	—	—
Trimipramine	99	—	—	1.5	—	—
Flunarizine	105	—	—	19.05	—	—
Tadalafil	108	—	—	—	—	100 *μ*M IC50 preventing Spike protein binding to ACE2
Thalidomide	109	—	—	—	—	11 versus 23 median days SARS-CoV-2 negative conversion from admission, 18.5 *vs*. 30 days length hospital stay
Paroxetine	111	—	—	—	—	0.52 hazard ratio (death or intubation)
Ifenprodil	117	—	—	—	46.86	Mpro 39% bound (50 *μ*M)
Nebivolol	123	—	—	2.72	—	—
Doxazosin	133	—	—	—	—	74% relative risk reduction (death)
Levofloxacin	145	—	—	418.6	—	—
Teniposide	149	—	—	—	—	46.3 *μ*M IC50 Spike protein binding ACE2

In addition to these validations gathered from the literature, 30 candidates were evaluated by our collaborator, Ennaid Therapeutics, of which 11 displayed *in vitro* efficacy; a patent has been filed for their use (Samudrala et al., 2020).

**FIGURE 4:**
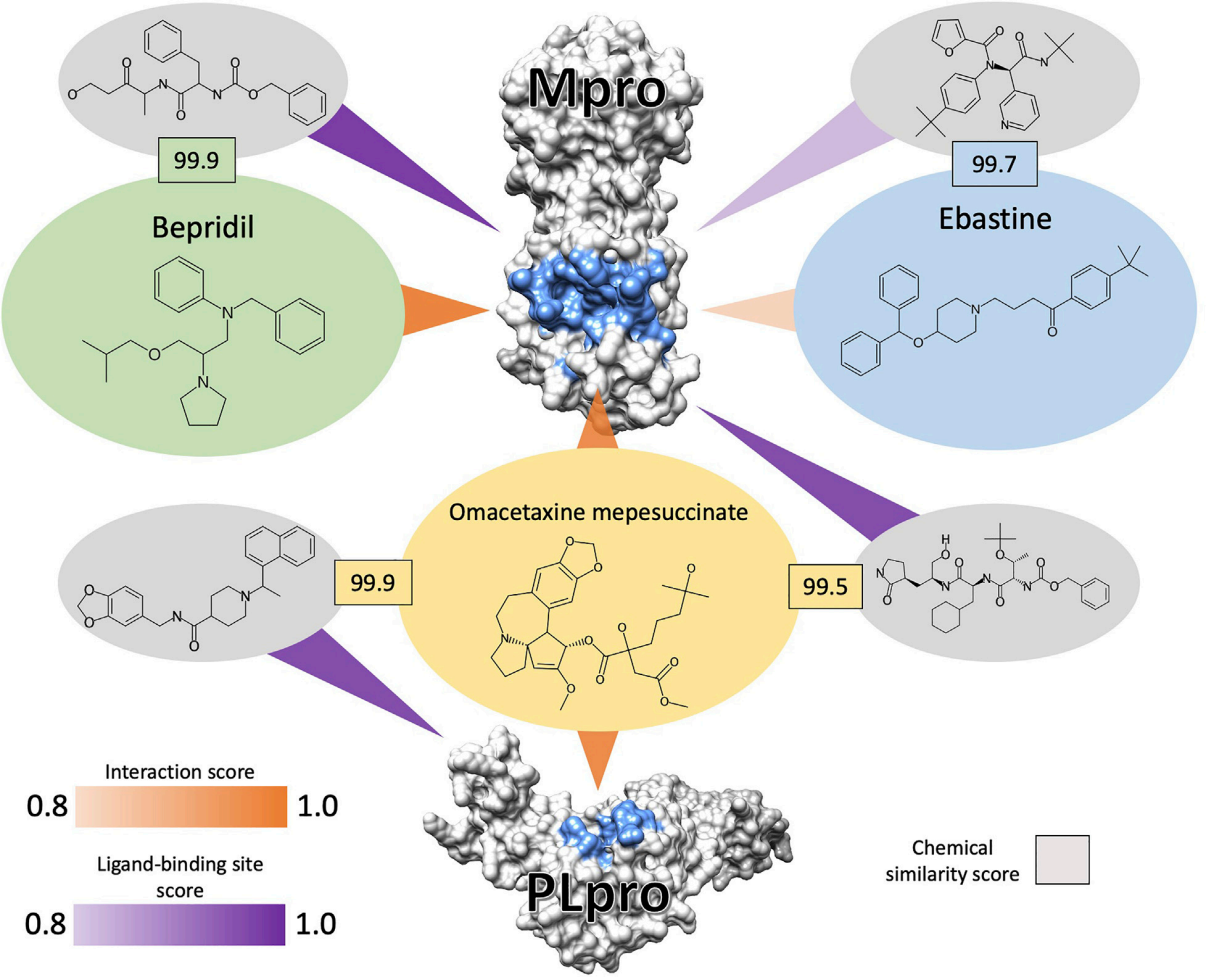
Analysis of selected interactions between SARS-CoV-2 proteases and top ranked CANDO-generated drug candidates. The main (Mpro, top) and the papain-like (PLpro, bottom) proteases are depicted in grey with the binding site residues colored blue. Bepridil (green) and omacetaxine mepesuccinate (orange) ranked at 15 and 1 in the 3.5.20 *de novo* list, and ebastine (blue) ranked at 92 in the 5.18.20 similarity list, are shown bound to one or both proteases. These constitute example interactions of when CANDO made a successful prediction as well as illustrate why candidate generation is not perfect from a mechanistic multiscale perspective. The interaction score (orange triangles) between the compounds and the proteases were generated using the bioanalytic docking protocol BANDOCK, with higher scores (maximum 1.0) predicting a higher likelihood of interaction. The ligand associated with the binding site predictions by the COACH algorithm and chosen as the template for BANDOCK are depicted in grey ellipses (full names available in the Supplementary Material), all of which are strong coronavirus protease inhibitors. These ligands are compared to the query drug using the ECFP10 chemical fingerprint *via* RDKit and a similarity score is assessed based on the Sorenson-Dice coefficient. The percentile of the similarity (black outlined boxes) from the corresponding distribution of all similarities between the query compounds and all ligands in the binding site library is multiplied by the confidence score associated with the binding site prediction from COACH (purple triangles) to serve as the final score. Bepridil inhibits the full SARS-CoV-2 virus and Mpro *in vitro* with EC50s of 0.86 and 72 μM, which was successfully assigned a strong interaction score of 0.98. On the other hand, ebastine also inhibits the full virus and Mpro with EC50s of 0.5 and 57 μM, yet was assigned a lower interaction score of 0.82. Despite the strong percentile similarity score between ebastine and its template ligand (99.7), the confidence score for this binding site prediction was 0.82, significantly lowering the final interaction score. However, ebastine is the 3rd most similar compound to nelfinavir, a known human immunodeficiency virus protease inhibitor with activity against SARS-CoV-2, based on interaction similarity to a library of 5,317 human proteins, suggesting its putative mechanism as a protease inhibitor. Omacetaxine mepesuccinate, the second strongest full virus inhibitor predicted by CANDO with an EC50 of 0.03 μM, was the top candidate from the *de novo* list and has interaction scores of 0.960 and 0.964 with Mpro and PLpro, respectively, and has not yet been validated in terms of target specificity. Based on the high interaction scores, we propose this as its mechanism not only for SARS-CoV-2, but for all other coronavirus species against which it has activity. In this manner, the mechanistic understanding of drug candidate behavior is readily deciphered in a multiscale manner, from the atomic-level fingerprints between the novel drug candidates and the interacting ligands to the evolutionary information embedded at the protein and proteome scales, and exemplifies the ability of the CANDO platform to accurately identify novel drug candidates and their mechanisms *via* a multi-pronged approach.

Aside from moxifloxacin and diphenhydramine, all validations of candidates ranked in the top 50 of their respective lists have full virus EC50 values less than 10 *μ*M. The same is true for those in the top 100 with the exception of hydroxyzine and terconazole. The second strongest reported EC50 (0.03 *μ*M) was obtained using omacetaxine mepesuccinate, the top ranked candidate from the 3.5.20 *de novo* list, which is only slightly weaker than the best EC50 belonging to ouabain (0.024 *μ*M), ranked 56 in the 5.18.20 similarity list. Figure 4 illustrates the proposed mechanism of omacetaxine mepesuccinate inhibiting SARS-CoV-2 *via* strong predicted interactions to the main and papain-like proteases. Two other drugs known to inhibit both SARS-CoV-2 proper as well as its main protease, bepridil and ebastine, were present in the 3.5.20 *de novo* and 5.18.20 similarity lists respectively, with the latter having a relatively weak interaction score to the main protease of 0.82 while the former received a score of 0.98. However, the protease inhibition activity of ebastine is supported by it being the third most similar compound to nelfinavir, a known human immunodeficiency virus protease inhibitor, based on their proteomic interaction signature similarity, suggesting the CANDO platform is capable of recognizing/predicting mechanistic behavior in multiple ways.

**FIGURE 5:**
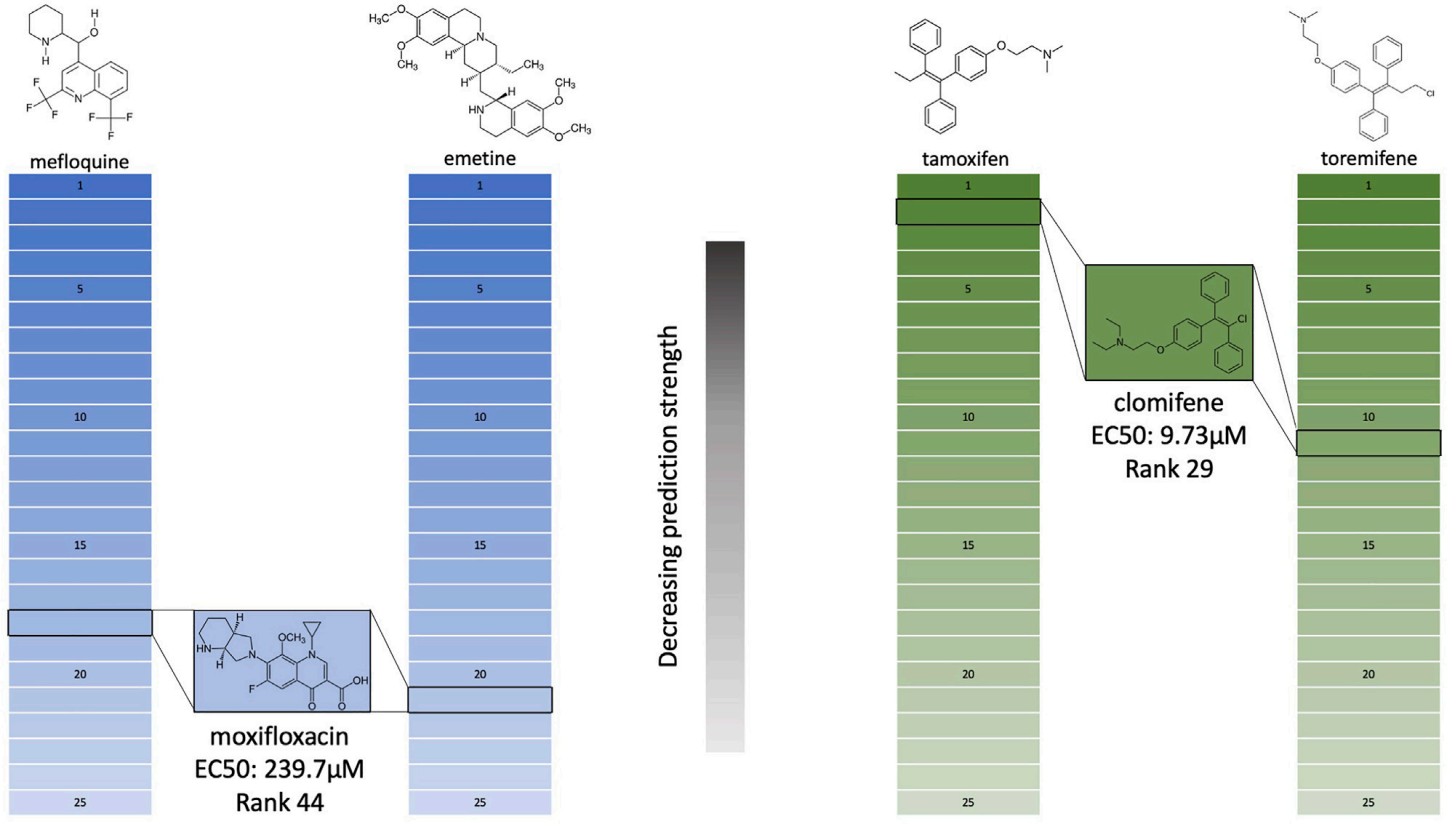
Analysis of the efficacy of two SARS-CoV-2 inhibitors with respect to proteomic interaction signature similarities predicted using the CANDO platform. The structures of two validated compounds from the 3.5.20 similarity list, moxifloxacin (blue) and clomifene (green), are shown with EC50 values of 239.7 and 9.73 μM, respectively. The EC50 values are based on the full virus *in vitro* inhibition of SARS-CoV-2. Their ranks in the list of the top 25 most similar compounds to two different coronavirus actives are outlined in black; moxifloxacin is at rank 18 and 21 in comparison to mefloquine and emetine, and clomifene is at rank 2 and 11 in comparison to tamoxifen and toremifene, respectively. These ranks are determined by the similarity coefficient (Sorenson-Dice) of the proteomic interaction signatures between the query compound and all others in the CANDO library. The proteomic signatures are vectors of interaction scores between a compound and a library of 5,317 human proteins computed using our in-house docking protocol BANDOCK (see Section 3.3). The fundamental hypothesis underlying the CANDO platform is that similar drugs will have similar behavior in biological systems as measured by their proteomic interaction signatures. Despite the relatively high rank (44) of moxifloxacin in the 3.5.20 similarity list, its measured EC50 was poor; this is explained by its lower interaction signature similarity to the two coronavirus actives depicted suggesting behavioral signal strength inversely correlates with rank. On the other hand, clomifene, the next highest prediction from the 3.5.20 similarity list at rank 29, has a stronger EC50 and ranks higher in the similarity lists to two coronavirus active compounds. However the reported EC50 values of mefloquine and emetine are strong at 4–15 and 0.46 *μ*M, respectively, which implies that behavioral similarity signal is preserved for highly ranked compounds and that using lower rank cutoff thresholds produces stronger candidates.

We also investigated why moxifloxacin was deemed a candidate despite its low reported efficacy (Figure 5). Moxifloxacin was predicted by the 3.5.20 similarity pipeline and received a score of two meaning it was in the top 25 most similar compounds to two coronavirus actives (average rank 19.5). Moxifloxacin was the 18th most similar compound to mefloquine and the 21st most similar to emetine; the former is a treatment for malaria, similar to many other anti-malarials with moderate activity (∼4–15 μM) against coronaviruses *in vitro* (Dyall et al., 2014; Ellinger et al., 2021), and the latter is an experimental treatment for amoebiasis with demonstrated activity against not only SARS-CoV-2 (EC50 0.46 μM) (Choy et al., 2020), but many other coronavirus species (Dyall et al., 2014; Shen et al., 2019). Moxifloxacin having similarity to one strong and one moderate anti-coronavirus compound would suggest a stronger EC50 than 239.7 μM; we attribute this result to a progressive decrease in behavioral/functional similarity signal strength/relevance as the distance between their proteomic interaction signatures relative to those of known coronavirus actives increases. In other words, the signal disappears as we move further down the ranks as depicted in Figure 5.

The second to last validation in the 3.5.20 similarity list is clomifene, an infertility treatment in women, at rank 29 with a score of 2 and EC50 of 9.73 μM; it is similar to the coronavirus active compounds tamoxifen (rank 2) and toremifene (rank 11), constituting an average rank of 6.5. Additionally, all other validations from the same list have an average rank of less than or equal to 6.5 regardless of the score, which ranges from two to six. This implies setting the cutoff rank for the canpredict module to a lower value will produce stronger candidates and is further supported by the higher hit rate observed in the 5.18.20 similarity list (29.9 *vs*. 24.4% for 3.5.20 similarity) which was produced with a cutoff of ten. However the candidates predicted in the 5.18.20 similarity list benefited from using anti-SARS-CoV-2 drugs specifically, as opposed to actives against other coronavirus species, and had over double the number of active compounds when compared to the actives used to generate the 3.5.20 similarity list.

The candidates generated using the human proteome interaction signature similarity pipeline had higher validation rates relative to the direct compound-protein inhibition *de novo* pipeline; yet some of the candidates generated by the latter demonstrated stronger *in vitro* efficacy. The increase in hit rate is due to the similarity pipeline utilizing the structural knowledge embedded in the results of countless coronavirus studies, whereas the *de novo* pipeline relies exclusively on the fidelity of the compound-protein interactions computed using our interaction scoring protocols, which are prone to inaccuracies. The *de novo* pipeline was better tuned to correctly rank the strong inhibitors as interaction scoring parameters were first optimized for SARS-CoV using the discounted cumulative gain metric, which prioritizes ranking the strongest active compounds near the top of the list. This suggests that weighting the active compounds based on their available EC50 values for the full proteome interaction similarity pipeline may produce more potent candidates.

Our observed hit rate of 18.5% is likely conservative as not all of the compounds from the three candidate lists have been validated for efficacy against SARS-CoV-2 in published clinical and experimental studies. Conversely, the fraction of these 51 validations analyzed in this study that will result in clinical utility is limited due to a variety of factors such as pharmacokinetics, pharmacodynamics, safety, and cost. Multiple candidates that we listed as validations, specifically chloroquine, hydroxychloroquine, and azithromycin, have had conflicting reports of clinical benefit (Wang Y. et al., 2020; Chowdhury et al., 2020; Spinner et al., 2020; Echeverría-Esnal et al., 2021); regardless, we consider them a successful prediction of the CANDO platform due to the extensive number of *in vitro* studies reporting their SARS-CoV-2 inhibition, which is what the compound-proteome interaction analytics pipelines present in CANDO optimize for at present. Furthermore, even if CANDO fails to accurately score a known interaction with our bioanalytic docking protocol (BANDOCK) for a compound with reported activity, as in the case of ebastine and the SARS-CoV-2 main protease, its therapeutic mechanism may still be elucidated by inspecting the behavior of highly similar compounds based on their proteomic interaction signatures. Consequently, we are actively implementing methods to further refine the feasibility of our candidates based on the aforementioned factors.

## 3 Methods

### 3.1 Compound structure library and known actives curation

The CANDO v2.1 compound library consisted of 8,696 drug and drug-like small molecule three-dimensional structures, including 1,979 approved for human use, and was extracted from DrugBank (Wishart et al., 2018); this library was used for the initial predictions. We later updated the CANDO compound library to v2.3 that included 13,194 compounds from DrugBank consisting of 2,449 approved drugs and 2,519 small molecule metabolites, with the remaining classified as experimental/investigational. Biologic therapeutics were not included in our analyses.

Initially, compounds were considered as a coronavirus active if they were identified in one of two high-throughput screens by Shen et al. and Dyall et al. (Dyall et al., 2014; Shen et al., 2019). The former screened a library of 290 compounds against SARS-CoV and Middle East respiratory syndrome coronavirus (MERS-CoV). The latter screened a 2,000 compound library against four different coronavirus strains: human coronavirus OC43 (HCoV-OC43), human coronavirus NL63 (HCoV-NL63), MERS-CoV, and murine coronavirus (MHV-A59; also known as mouse hepatitis virus). Out of 60 successful hits from both studies, 18 compounds from the Shen study along with their EC50s against HCoV-OC43 and HCoV-NL63, as well as 12 compounds from the Dyall study and their EC50s against SARS-CoV were mapped to our compound library. These three actives sublibraries were used for the compound-protein interaction scoring protocol parameter optimization (see Section 3.4).

The nonredundant combination of actives in the Shen and Dyall studies were used for the signature similarity candidate generation pipeline (see Section 3.5). We also added oseltamivir and remdesivir as at that time (February 2020) evidence suggested that they may inhibit SARS-CoV-2 or related coronaviruses (Wang M. et al., 2020; Coenen et al., 2020), resulting in an actives library of 38 compounds.

As more data became available regarding *in vitro* efficacy values for compounds against SARS-CoV-2, a second sublibrary of 85 actives with reported EC50 values less than or equal to 10 *μ*M was extracted on May 7, 2020 from the Targeting COVID-19 Portal from GHDDI (Leng, 2020), which contained 17/38 compounds from the previous list. The updated CANDO compound library along with the new GHDDI actives sublibrary were used for the enhanced signature similarity candidate generation pipeline (see Section 3.5).

### 3.2 Protein structure library curation

The available SARS-CoV x-ray diffraction protein structures were obtained from the Protein Data Bank (PDB) (Burley et al., 2019) and initially served as our representative coronavirus proteome, comprising eighteen total structures. These eighteen SARS-CoV proteins were used for the compound-protein interaction protocol optimization (see Section 3.3).

A SARS-CoV-2 protein library of 24 structures was modeled from sequence using the I-TASSER v5.1 suite (Yang et al., 2015) and comprised the proteome used for the remaining analyses. We prioritized 18/24 proteins that were modeled by I-TASSER using homology to known coronavirus structures. These 18 SARS-CoV-2 proteins were used for the *de novo* pipeline, while both iterations of the signature similarity based pipeline (see Section 3.5) used a library of 5,317 human protein x-ray diffraction structures extracted from the PDB. The former piepline is implemented using the canpredict *de novo* module, and the latter is implemented using the canpredict similarity module, in the cando.py Python package (Mangione et al., 2020a; Mangione and Falls, 2022)).

### 3.3 Compound-protein interaction calculation

We utilized our in-house bioinformatic analytics-based docking protocol BANDOCK to generate interaction scores between every compound and every protein structure; these scores serve as a proxy for binding strength/probability (Minie et al., 2014; Sethi et al., 2015; Falls et al., 2019; Hudson and Samudrala, 2021). The COACH algorithm from the I-TASSER suite (Yang et al., 2013) was used to predict binding sites for each protein. COACH outputs an associated score and binding ligand for every binding site in a protein and is the primary data used by BANDOCK to generate interaction scores. For a given compound and protein pair, every interacting ligand predicted by COACH is compared to the query compound by computing the similarity coefficient of their chemical fingerprints generated *via* RDKit (Landrum, 2013). The maximum resulting coefficient (i.e. the strongest match) and its associated binding site score are then used to compute the final interaction score for the compound-protein pair, depending on the scoring protocol parameters. This is repeated iteratively for each protein in a given library (e.g. SARS-CoV, SARS-CoV-2, human, nonredundant PDB), resulting in a proteomic interaction signature for every drug/compound, represented an *N* × *M* matrix, where *N* is the number of drugs/compounds and *M* is the number of proteins.

Interaction scoring (BANDOCK) parameters were systematically varied to identify those optimal for assessing anti-coronavirus activity. These were 1) the chemical fingerprinting method: ECFP or functional-class fingerprint (FCFP) with diameters of 0, 2, 4, 6, 8, and 10 and length of 2048; 2) the fingerprint style: binary vs integer based for the compounds/ligands; 3) the scoring protocol: the binding site score from COACH (Pscore), the Tanimoto or Sorenson-Dice coefficient of the binding site ligand from COACH to the query drug (Cscore) for binary or integer fingerprints, respectively, the percentile of the Cscore in the distribution of all I-TASSER ligand Cscores to the query drug (dCscore), or products of these (Pscore × Cscore, Pscore × dCscore); and 4) thresholds: Pscore and Cscore (or dCscore) thresholds so that any binding site or compound-ligand similarity coefficient (or its percentile) that does not exceed each cutoff, respectively, are ignored. A compound-protein interaction matrix was generated for each of these parameter combinations.

Computed interaction scores with the 18 SARS-CoV proteins were used for compound-protein scoring protocol parameter optimization, while the scores computed (using the parameters identified in the previous step) with the 18 SARS-CoV-2 proteins were used for the *de novo* candidate generation pipeline. The scores computed with a library of 5,317 human PDB structures were used for the similarity-based pipelines (see section 3.5). The initial parameters were an ECFP4 binary fingerprint with Tanimoto coefficients for Cscores, Pscore scoring protocol, and a dCscore threshold of 0.5 (50th percentile), which were used to generate the March 5 2020 aka 3.5.20 list of candidates. The enhanced parameters were an ECFP4 integer fingerprint with Sorenson-Dice coefficient for Cscores, Pscore × dCscore scoring protocol, and a dCscore threshold of 0.75 (75th percentile), which were used to generate the May 18, 2020 aka 3.18.20 candidate list.

### 3.4 Parameter optimization using coronavirus active compound recovery

We identified the best parameters for BANDOCK that optimally ranked the compounds identified *via* high throughput screens against three different coronavirus species (SARS-CoV, HCoV-NL63, and HCoV-OC43), each of which were assessed separately *via de novo* drug candidate generation. We also varied the cutoff threshold of interaction scores to consider so that the interaction scores with proteins below that threshold were not considered in the total for a given compound. The cutoffs in this study were incremented by 0.05, starting with 0.0 (no threshold) and ending with 1.0 (maximum score). The discounted cumulative gain metric (Järvelin and Kekäläinen, 2002; Dupret, 2011), often employed for search engine optimization and other early recognition problems, was used to assess how well each matrix properly ranked the active compounds in the proper order given their associated EC50/IC50 values from each of the three species separately. Our previous work has identified this metric as the optimal one for drug repurposing studies (Schuler et al., 2021). Briefly, discounted cumulative gain (DCG) rewards lists of predictions that rank the optimal known actives at the top and progressively penalizes lower ranked ones *via* the equation:

$$DCG_{p}=\overset{p}{\underset{i=1}{\sum }}\frac{2^{rel_{i}}-1}{\log_{2}\left(i+1\right)}$$ (1)

where *p* is the length of the list, *i* is the rank, and *rel*_*i*_ is the relevance score of the item at position/rank *i* which is the inverse of the EC50 values (1/EC50) for the 36 nonredundant actives.

Parameter sets utilizing any of the following criteria were discarded due to trivial candidate rankings: Pscore scoring protocol, interaction score threshold of 1.0, and Cscore threshold of 1.0. Interaction scores generated using the Pscore protocol did not utilize the chemical fingerprint similarity value between the binding site ligand and the query compound and subsequently failed to discriminate between two compounds that used the same ligand. Using an interaction score or Cscore threshold of 1.0 required the chemical fingerprint similarity score to equal 1.0, meaning identical compounds, therefore ensuring the only predicted candidates were known coronavirus inhibitors.

### 3.5 COVID-19 drug candidate generation

To generate drug candidates against COVID-19, we used both a *de novo* pipeline that ranked compounds based on their predicted interaction scores against proteins from SARS-CoV-2, and a similarity pipeline that searched the CANDO drug/compound library for compounds similar to those deemed as actives in terms of their interaction signatures. The former protocol summed the computed interaction scores of each compound against all viral proteins and ranked them from best to worst. Interaction scores below particular thresholds were ignored in the sums (see section 3.4). For the initial iteration of the latter similarity protocol, drug candidates were ranked by their frequency of occurrence in the top 25 most similar compounds to each of the 38 coronavirus actives, while the enhanced iteration ranked compounds by frequency of occurrence in the top 10 most similar compounds to the 85 GHDDI actives. We kept track of the number of coronavirus actives each compound was similar to within the cutoff threshold along with their average ranks (which served as a tie-breaker) to produce the final ranked list of candidates.

The outputs of our pipelines were three ranked lists of drug candidates: one using the direct viral inhibition pipeline from the initial iteration (3.5.20 *de novo*), a second using the similarity based candidate generation pipeline from the initial iteration (3.5.20 similarity), and the third using the similarity based pipeline using the enhanced actives list (5.18.20 similarity).

### 3.6 External validation studies curation

We analyzed GHDDI (Leng, 2020) and CoronaCentral (Lever and Altman, 2021) for up-to-date information on COVID-19 therapeutic interventions which could independently and prospectively validate our top ranked candidates. Both sources utilize deep learning or natural language processing methods to automatically extract and annotate information from SARS-CoV-2 studies to produce lists of possible actives. We manually parsed the manuscripts that were annotated with and matched the name of any candidate compounds from our three prediction lists for corresponding efficacy values (EC50, IC50, hazard ratios, etc) while eliminating studies that were purely computational or did not investigate the candidate compound as the primary intervention.

## 4 Conclusion

This study highlights how CANDO may be used to rapidly generate promising leads for drug development when time is critical, provided the therapeutic intervention is possible within established dosing guidelines. Our study is an assessment of potential therapeutics for treating COVID-19 which were all generated within three months of the pandemic declaration by the WHO. Considering that it took almost one year for a vaccine (Food and Administration, 2022) and two years for a potent antiviral such as molnupiravir or nirmatrelvir (Mahase, 2021; Hammond et al., 2022) to become available, we have exemplified that computational drug discovery and repurposing platforms like ours can be strategically used to alleviate the burden of emergent pathogens ahead of time. Additional studies, ideally *via in vivo* and/or clinical studies, verifying the efficacy of these identified candidates is necessary in most cases, however for already approved drug candidates such as those explored in this study the need for trials demonstrating safety is greatly diminished. Additionally, retrospective EHR analysis may also be used to indirectly examine clinical benefits in human patients as in the case of fluoxetine (Oskotsky et al., 2021).

## Data Availability

The datasets presented in this study can be found in online repositories. The names of the repository/repositories and accession number(s) can be found below: http://compbio.buffalo.edu/data/mc_cando_covid19/.
