# Supplementary material for: Optimal COVID-19 therapeutic candidate discovery using the CANDO platform
Source: Front Pharmacol. 2022 Aug 25;13:970494. doi: 10.3389/fphar.2022.970494 (PMC9452636; doi:10.3389/fphar.2022.970494)
Supplement: Supplementary file 1 [file DataSheet1.pdf]

Supplementary Material

REFERENCES

Bafna, K., White, K., Harish, B., Rosales, R., Ramelot, T. A., Acton, T. B., et al. (2020). Hepatitis c virus drugs simeprevir and grazoprevir synergize with remdesivir to suppress sars-cov-2 replication in cell culture. *bioRxiv*

Bakowski, M. A., Beutler, N., Chen, E., Nguyen, T.-T. H., Kirkpatrick, M. G., Parren, M., et al. (2020). Oral drug repositioning candidates and synergistic remdesivir combinations for the prophylaxis and treatment of covid-19. *BioRxiv*

Bocci, G., Bradfute, S. B., Ye, C., Garcia, M. J., Parvathareddy, J., Reichard, W., et al. (2020). Virtual and in vitro antiviral screening revive therapeutic drugs for covid-19. *ACS Pharmacology & Translational Science* 3, 1278–1292

Cagno, V., Magliocco, G., Tapparel, C., and Daali, Y. (2020). The tyrosine kinase inhibitor nilotinib inhibits sars-cov-2 in vitro. *Basic & Clinical Pharmacology & Toxicology*

Carpinteiro, A., Edwards, M. J., Hoffmann, M., Kochs, G., Gripp, B., Weigang, S., et al. (2020). Pharmacological inhibition of acid sphingomyelinase prevents uptake of sars-cov-2 by epithelial cells. *Cell Reports Medicine* 1, 100142

Chen, C. Z., Xu, M., Pradhan, M., Gorshkov, K., Petersen, J. D., Straus, M. R., et al. (2020). Identifying sars-cov-2 entry inhibitors through drug repurposing screens of sars-s and mers-s pseudotyped particles. *ACS Pharmacology & Translational Science* 3, 1165–1175

Chiou, W.-C., Hsu, M.-S., Chen, Y.-T., Yang, J.-M., Tsay, Y.-G., Huang, H.-C., et al. (2021). Repurposing existing drugs: identification of sars-cov-2 3c-like protease inhibitors. *Journal of Enzyme Inhibition and Medicinal Chemistry* 36, 147–153

Cho, J., Lee, Y. J., Kim, J. H., il Kim, S., Kim, S. S., Choi, B.-S., et al. (2020). Antiviral activity of digoxin and ouabain against sars-cov-2 infection and its implication for covid-19. *Scientific reports* 10, 1–8

Choy, K.-T., Wong, A. Y.-L., Kaewpreedee, P., Sia, S. F., Chen, D., Hui, K. P. Y., et al. (2020). Remdesivir, lopinavir, emetine, and homoharringtonine inhibit sars-cov-2 replication in vitro. *Antiviral research* 178, 104786

De Meyer, S., Bojkova, D., Cinatl, J., Van Damme, E., Buyck, C., Van Loock, M., et al. (2020). Lack of antiviral activity of darunavir against sars-cov-2. *International Journal of Infectious Diseases* 97, 7–10

Drayman, N., Jones, K. A., Azizi, S.-A., Froggatt, H. M., Tan, K., Maltseva, N. I., et al. (2020). Drug repurposing screen identifies masitinib as a 3clpro inhibitor that blocks replication of sars-cov-2 in vitro. *bioRxiv*

Ellinger, B., Bojkova, D., Zaliani, A., Cinatl, J., Claussen, C., Westhaus, S., et al. (2021). A sars-cov-2 cytopathicity dataset generated by high-content screening of a large drug repurposing collection. *Scientific data* 8, 1–10

Fintelman-Rodrigues, N., Sacramento, C. Q., Ribeiro Lima, C., Souza da Silva, F., Ferreira, A. C., Mattos, M., et al. (2020). Atazanavir, alone or in combination with ritonavir, inhibits sars-cov-2 replication and proinflammatory cytokine production. *Antimicrobial agents and chemotherapy* 64, e00825–20

Gammeltoft, K. A., Zhou, Y., Duarte Hernandez, C. R., Galli, A., Offersgaard, A., Costa, R., et al. (2020). Hepatitis c virus protease inhibitors show differential efficacy and interactions with remdesivir for treatment of sars-cov-2 in vitro. *Antimicrobial Agents and Chemotherapy* , AAC–02680

Garcia Jr, G., Sharma, A., Ramaiah, A., Sen, C., Kohn, D. B., Gomperts, B. N., et al. (2020). Antiviral drug screen of kinase inhibitors identifies cellular signaling pathways critical for sars-cov-2 replication. *Available at SSRN 3682004*

Gupta, A., Rani, C., Pant, P., Vijayan, V., Vikram, N., Kaur, P., et al. (2020). Structure-based virtual screening and biochemical validation to discover a potential inhibitor of the sars-cov-2 main protease. *ACS omega*

Günther, S., Reinke, P. Y., Fernández-García, Y., Lieske, J., Lane, T. J., Ginn, H. M., et al. (2021). X-ray screening identifies active site and allosteric inhibitors of sars-cov-2 main protease. *Science* 372, 642–646

Hoertel, N., Sánchez, M., Vernet, R., Beeker, N., Neuraz, A., Blanco, C., et al. (2020). Association between hydroxyzine use and reduced mortality in patients hospitalized for coronavirus disease 2019: results from a multicenter observational study. *medRxiv*

Hoertel, N., Sánchez-Rico, M., Vernet, R., Beeker, N., Jannot, A.-S., Neuraz, A., et al. (2021a). Association between antidepressant use and reduced risk of intubation or death in hospitalized patients with covid-19: results from an observational study. *Molecular psychiatry* , 1–14

Hoertel, N., Sánchez-Rico, M., Vernet, R., Jannot, A.-S., Neuraz, A., Blanco, C., et al. (2021b). Observational study of chlorpromazine in hospitalized patients with covid-19. *Clinical drug investigation* 41, 221–233

Ianevski, A., Yao, R., Fenstad, M. H., Biza, S., Zusinaite, E., Reisberg, T., et al. (2020). Potential antiviral options against sars-cov-2 infection. *Viruses* 12, 642

Kato, F., Matsuyama, S., Kawase, M., Hishiki, T., Katoh, H., and Takeda, M. (2020). Antiviral activities of mycophenolic acid and imd-0354 against sars-cov-2. *Microbiology and Immunology* 64, 635–639

Ko, M., Jeon, S., Ryu, W.-S., and Kim, S. (2020). Comparative analysis of antiviral efficacy of fda-approved drugs against sars-cov-2 in human lung cells. *Journal of medical virology*

Konrat, R., Papp, H., Szijarto, V., Gesell, T., Nagy, G., Madai, M., et al. (2020). The anti-histamine azelastine, identified by computational drug repurposing, inhibits sars-cov-2 infection in reconstituted human nasal tissue in vitro. *bioRxiv*

Li, Y., Shi, K., Qi, F., Yu, Z., Chen, C., Pan, J., et al. (2021). Thalidomide combined with short-term low-dose glucocorticoid therapy for the treatment of severe covid-19: a case-series study. *International Journal of Infectious Diseases* 103, 507–513

Lo, H. S., Hui, K. P. Y., Lai, H.-M., He, X., Khan, K. S., Kaur, S., et al. (2021). Simeprevir potently suppresses sars-cov-2 replication and synergizes with remdesivir. *ACS central science* 7, 792–802

Mahdi, M., Mótyán, J. A., Szojka, Z. I., Golda, M., Miczi, M., and Tőzsér, J. (2020). Analysis of the efficacy of hiv protease inhibitors against sars-cov-2's main protease. *Virology journal* 17, 1–8

Manandhar, A., Blass, B. E., Colussi, D. J., Almi, I., Abou-Gharbia, M., Klein, M. L., et al. (2021). Targeting sars-cov-2 m3clpro by hcv ns3/4a inhibitors: In silico modeling and in vitro screening. *Journal of chemical information and modeling* 61, 1020–1032

Pickard, A., Calverley, B. C., Chang, J., Garva, R., Lu, Y., and Kadler, K. E. (2021). Discovery of re-purposed drugs that slow sars-cov-2 replication in human cells. *bioRxiv*

Pizzorno, A., Padey, B., Dubois, J., Julien, T., Traversier, A., Dulière, V., et al. (2020). In vitro evaluation of antiviral activity of single and combined repurposable drugs against sars-cov-2. *Antiviral research* 181, 104878

Plaze, M., Attali, D., Prot, M., Petit, A.-C., Blatzer, M., Vinckier, F., et al. (2021). Inhibition of the replication of sars-cov-2 in human cells by the fda-approved drug chlorpromazine. *International journal of antimicrobial agents* 57, 106274

Reznikov, L. R., Norris, M. H., Vashisht, R., Bluhm, A. P., Li, D., Liao, Y.-S. J., et al. (2021). Identification of antiviral antihistamines for covid-19 repurposing. *Biochemical and biophysical research communications* 538, 173–179

Rivas, M. D., Saponi-Cortes, J. M. R., and Zamorano, J. (2021). Hydroxyzine inhibits sars-cov-2 spike protein binding to ace2 in a qualitative in vitro assay. *bioRxiv*

Rose, L., Graham, L., Koenecke, A., Powell, M., Xiong, R., Shen, Z., et al. (2021). The association between alpha-1 adrenergic receptor antagonists and in-hospital mortality from covid-19. *Frontiers in Medicine* 8

Schloer, S., Brunotte, L., Goretzko, J., Mecate-Zambrano, A., Korthals, N., Gerke, V., et al. (2020). Targeting the endolysosomal host-sars-cov-2 interface by clinically licensed functional inhibitors of acid sphingomyelinase (fiasma) including the antidepressant fluoxetine. *Emerging Microbes & Infections* 9, 2245–2255

Schloer, S., Brunotte, L., Mecate-Zambrano, A., Zheng, S., Tang, J., Ludwig, S., et al. (2021). Drug synergy of combinatory treatment with remdesivir and the repurposed drugs fluoxetine and itraconazole effectively impairs sars-cov-2 infection in vitro. *British Journal of Pharmacology* 178, 2339–2350

Scroggs, S. L., Offerdahl, D. K., Flather, D. P., Morris, C. N., Kendall, B. L., Broeckel, R. M., et al. (2021). Fluoroquinolone antibiotics exhibit low antiviral activity against sars-cov-2 and mers-cov. *Viruses* 13, 8

Si, L., Bai, H., Rodas, M., Cao, W., Oh, C. Y., Jiang, A., et al. (2020). Human organ chip-enabled pipeline to rapidly repurpose therapeutics during viral pandemics. *bioRxiv*

Touret, F., Gilles, M., Barral, K., Nougairède, A., van Helden, J., Decroly, E., et al. (2020). In vitro screening of a fda approved chemical library reveals potential inhibitors of sars-cov-2 replication. *Scientific reports* 10, 1–8

Tsegay, K. B., Adeyemi, C. M., Gniffke, E. P., Sather, D. N., Walker, J. K., and Smith, S. E. P. (2021). A repurposed drug screen identifies compounds that inhibit the binding of the covid-19 spike protein to ace2. *Frontiers in Pharmacology* 12, 1385. doi:10.3389/fphar.2021.685308

Tsiakos, K., Tsakiris, A., Tsibris, G., Voutsinas, P., Panagopoulos, P., Kosmidou, M., et al. (2021). Early start of oral clarithromycin is associated with better outcome in covid-19 of moderate severity: the achieve open-label trial. *medRxiv* , 2020–12

Van Damme, E., De Meyer, S., Bojkova, D., Ciesek, S., Cinatl, J., De Jonghe, S., et al. (2021). In vitro activity of itraconazole against sars-cov-2. *Journal of Medical Virology* 93, 4454–4460

Vatansever, E. C., Yang, K. S., Drelich, A. K., Kratch, K. C., Cho, C.-C., Kempaiah, K. R., et al. (2021). Bepridil is potent against sars-cov-2 in vitro. *Proceedings of the National Academy of Sciences* 118

Weston, S., Coleman, C. M., Haupt, R., Logue, J., Matthews, K., Li, Y., et al. (2020). Broad anti-coronavirus activity of food and drug administration-approved drugs against sars-cov-2 in vitro and sars-cov in vivo. *Journal of virology* 94, e01218–20

Xiong, H.-L., Cao, J.-L., Shen, C.-G., Ma, J., Qiao, X., Shi, T., et al. (2020). Several fda-approved drugs effectively inhibit sars-cov-2 infection in vitro. *bioRxiv*

Yang, L., Pei, R.-j., Li, H., Ma, X.-n., Zhou, Y., Zhu, F.-h., et al. (2020). Identification of sars-cov-2 entry inhibitors among already approved drugs. *Acta Pharmacologica Sinica* , 1–7

Yuan, Z., Pavel, M. A., Wang, H., and Hansen, S. B. (2020). Hydroxychloroquine: mechanism of action inhibiting sars-cov2 entry. *bioRxiv*

Table S1. Complete list of validated candidates generated by the CANDO platform including references.

Compound	Ref.
omacetaxine mepesuccinate	Ianevski et al. (2020)
chlorpromazine	Weston et al. (2020); Plaze et al. (2021); Hoertel et al. (2021b)
clomipramine	Weston et al. (2020)
entrectinib	Tsegay et al. (2021)
mycophenolate mofetil	Kato et al. (2020)
imipramine	Schloer et al. (2020); Carpinteiro et al. (2020)
toremifene	Yang et al. (2020); Weston et al. (2020)
tamsulosin	Rose et al. (2021)
bepridil	Vatansever et al. (2021)
azelastine	Drayman et al. (2020); Yang et al. (2020); Reznikov et al. (2021); Konrat et al. (2020)
zuclopenthixol	Bocci et al. (2020)
masitinib	Drayman et al. (2020)
erythromycin	Yuan et al. (2020)
chloroquine	Ko et al. (2020)
ritonavir	Mahdi et al. (2020)
hydroxychloroquine	Touret et al. (2020); Weston et al. (2020)
cobicistat	De Meyer et al. (2020); Gupta et al. (2020)
amodiaquine	Bocci et al. (2020); Ianevski et al. (2020); Weston et al. (2020); Si et al. (2020); Ko et al. (2020); Pickard et al. (2021)
nilotinib	Cagno et al. (2020); Garcia Jr et al. (2020); Tsegay et al. (2021)
pimozide	Vatansever et al. (2021)
diphenhydramine	Reznikov et al. (2021)
clomifene	Xiong et al. (2020)
remdesivir	Ellinger et al. (2021); Pizzorno et al. (2020); Ko et al. (2020)
butenafine	Chiou et al. (2021)
moxifloxacin	Scroggs et al. (2021)
clarithromycin	Tsiakos et al. (2021)
saquinavir	Mahdi et al. (2020); Vatansever et al. (2021); Chiou et al. (2021)
simeprevir	Manandhar et al. (2021); Lo et al. (2021); Bakowski et al. (2020); Bafna et al. (2020); Gammeltoft et al. (2020)
ouabain	Cho et al. (2020)
azithromycin	Touret et al. (2020)
tranylcypromine	Chiou et al. (2021)
almitrine	Ellinger et al. (2021)
tamoxifen	Weston et al. (2020)
colistimethate	Günther et al. (2021)
lopinavir	Ko et al. (2020); Ellinger et al. (2021); Vatansever et al. (2021); Choy et al. (2020)
terconazole	Weston et al. (2020)
silodosin	Rose et al. (2021)
atazanavir	Mahdi et al. (2020); Fintelman-Rodrigues et al. (2020)
triamterene	Tsegay et al. (2021)
hydroxyzine	Reznikov et al. (2021); Hoertel et al. (2020); Rivas et al. (2021)
itraconazole	Vatansever et al. (2021); Van Damme et al. (2021); Schloer et al. (2021)
ebastine	Vatansever et al. (2021); Pickard et al. (2021)
avatrombopag	Ellinger et al. (2021)
trimipramine	Chen et al. (2020); Drayman et al. (2020)
flunarizine	Ellinger et al. (2021)
tadalafil	Tsegay et al. (2021)
thalidomide	Li et al. (2021)
paroxetine	Hoertel et al. (2021a)
ifenprodil	Günther et al. (2021)
nebivolol	Bocci et al. (2020)
doxazosin	Rose et al. (2021)
levofloxacin	Scroggs et al. (2021)
teniposide	Tsegay et al. (2021)

Table S2. Stability of validated candidates generated by the CANDO platform. The compound energies were calculated using the obminimize function of the OpenBabel package with the General Amber Force Field (GAFF), maximum of 2,500 steps, convergence criteria of 1e-6, and conjugate gradient algorithm. The name of the compound and the final energy after geometrical optimization are given (all compounds reached convergence before the maximum number of steps).

Compound	Energy
omacetaxine mepesuccinate	316.093
chlorpromazine	169.016
clomipramine	178.924
entrectinib	309.601
mycophenolate mofetil	255.152
imipramine	179.616
toremifene	83.325
tamsulosin	180.825
bepridil	133.576
azelastine	111.516
zuclopenthixol	152.377
masitinib	121.758
erythromycin	1075.716
chloroquine	84.332
ritonavir	-23.182
hydroxychloroquine	93.528
cobicistat	88.753
amodiaquine	102.659
nilotinib	222.192
pimozide	241.414
diphenhydramine	50.734
clomifene	93.661
remdesivir	223.769
butenafine	62.797
clarithromycin	1124.801
saquinavir	227.095
simeprevir	306.779
ouabain	466.127
azithromycin	977.056
tranylcypromine	251.252
almitrine	509.492
tamoxifen	101.626
colistimethate	668.173
lopinavir	114.65
terconazole	187.172
silodosin	163.902
atazanavir	21.233
triamterene	798.561
hydroxyzine	113.081
itraconazole	220.249
ebastine	141.273
avatrombopag	277.992
trimipramine	174.978
flunarizine	109.818
tadalafil	253.428
thalidomide	70.096
paroxetine	188.704
ifenprodil	54.766
nebivolol	60.837
doxazosin	534.22
teniposide	326.968
